# Intratumoral Treatment with 5-Androstene-3β, 17α-Diol Reduces Tumor Size and Lung Metastasis in a Triple-Negative Experimental Model of Breast Cancer

**DOI:** 10.3390/ijms231911944

**Published:** 2022-10-08

**Authors:** Rocío Alejandra Ruiz Manzano, Karen Elizabeth Nava-Castro, Margarita Isabel Palacios-Arreola, Rosalía Hernández-Cervantes, Víctor Hugo Del Río-Araiza, Mariana Segovia-Mendoza, Armando Pérez-Torres, Manuel Iván Girón-Pérez, Jorge Morales-Montor

**Affiliations:** 1 Departamento de Inmunología, Instituto de Investigaciones Biomédicas, Universidad Nacional Autónoma de México, Ciudad de México 04510, Mexico; 2 Grupo de Biología y Química Atmosférica, Departamento de Ciencias Ambientales, Instituto de Ciencias de la Atmósfera, y Cambio Climático, Universidad Nacional Autónoma de México, Ciudad de México 04510, Mexico; 3 Departamento de Parasitología, Facultad de Medicina Veterinaria y Zootecnia, Universidad Nacional Autónoma de México, Ciudad de México 04510, Mexico; 4 Departamento de Farmacología, Facultad de Medicina, Universidad Nacional Autónoma de México, Ciudad de México 04510, Mexico; 5 Departamento de Biologia Celular y Tisular, Facultad de Medicina, Universidad Nacional Autónoma de México, Edificio A, 4to Piso, Ciudad Universitaria, Ciudad de México 04510, Mexico; 6 Laboratorio Nacional para la Investigación en Inocuidad Alimentaria, Unidad Nayarit, Universidad Autónoma de Nayarit, Tepic 63000, Mexico

**Keywords:** breast tumor, intratumoral treatment, alpha-androstenediol, translational medicine, metastasis, tumor microenvironment

## Abstract

Breast cancer treatment failure is related to low response rates, high costs, and long-term toxicities. Thus, it is necessary to find less toxic, cheaper, and more effective treatments. In situ administration ensures drug delivery to tumor cells and decreases systemic toxic effects. The androstene-3β, 17α-diol (α-AED) reduces breast tumor cell proliferation and is an ideal candidate to treat mammary tumors. This study aims to identify the in vitro and in vivo effects of α-AED on a triple-negative mammary tumor model. An in vitro biphasic steroid effect was observed in mouse and human mammary tumor cells treated with α-AED. In this sense, cells treated with higher doses (100 and 200 μM) showed an antiproliferative effect. The α-AED administrated intratumorally reduced average tumor weight and increased the percentage of natural killer cells (NK), plasmatic, and plasmablast cells in mice tumors. Of note, VEGF levels in all α-AED-treated tumors was lower than in the control and vehicle groups. The tumor in situ increased response was reflected systemically by higher anti-4T1 IgG concentration in serum from α-AED-treated mice, but no other associated systemic changes were detected. The reduction in tumor size for the local injection of α-AED is associated with the anti-proliferative effect of this steroid, and the lower local levels of VEGF may be related to the imperceptible macroscopic metastasis in α-AED-treated mice. The above suggests that α-AED may be used in clinical studies to prove its efficacy as an alternative breast tumor treatment or in conjunction with already established therapies.

## 1. Introduction

There is a broad range of accepted therapeutic approaches to treat breast cancer. The treatments employed in breast cancer aim to reduce cell proliferation, tumor growth, and the risk of metastasis development [1]. Among these therapies, estrogen antagonists, tamoxifen, and aromatase inhibitors are used as adjuvant therapy to treat hormone receptor-positive breast cancer. However, toxicity associated with these treatments is related to low response rates and long-term side effects [2]. Additionally, patients with triple-negative breast cancer (TNBC) do not benefit from anti-estrogenic therapy [3].

Aside from estrogenic antagonists, other steroid compounds, such as dehydroepiandrosterone (DHEA), have been proven to inhibit the proliferation and migration of breast cancer cell lines in vitro and prevent the development of breast cancer after mutagen administration [4,5]. Another steroid with a potent antiproliferative effect is the DHEA-analogue named 5-Androstene-3β, 17α-diol (α-AED). This hormone is more effective in inhibiting MCF-7 and MDA-MB231 breast cancer cell lines [4,6]. Regarding α-AED, its antiproliferative effect is independent of α- estrogen or androgen receptors [7]. 

Androstene α-AED is an epimer of 5-Androstene-3β, 17β-diol (α-AED), which has been shown to upregulate immune activity [8]. Both steroids occur naturally, α-AED being found to be secreted in the spermatic vein of human testes [9]. This steroid is also found in amniotic fluid and fetal-placenta circulation of normally-expected pregnancies. Low levels were related to pathological pregnancies linked to diabetes, toxemia, and placental insufficiency [10].

Even though androstenes are related to immune regulation, the main effect of α-AED is on the proliferation of tumor cells. Their effects have been widely studied in in vitro experiments to evaluate changes in immune system cells and the development of tumor cells, which have been done independently. Thus, the resulting interaction between the immune system and the cancer context remains unknown. The interaction aforementioned is relevant, knowing that immune cells influence the tumor’s microenvironment and can determine if the tumor development halts or progresses [11,12].

In addition to immune cell proportions and phenotypes, other important drivers in the tumor microenvironment are the soluble factors that play an essential role in the immune tumor milieu. In this sense, angiogenesis is associated with tumor nutrient supply and metastasis through the stimulation of the vascular endothelial growth factor (VEGF) [13]. Hence, elevated levels of VEGF correlate with an increase in lymph node metastasis and worse prognosis in patients with breast tumors [14]. All things considered, it is possible to directly modify the tumor microenvironment by directly administering pharmacological agents [15]. Thus, to address the local microenvironmental modifications of the tumor and immune cells by α-AED, this compound was administered directly into mice tumors induced by 4T1 mammary tumor cells. 

## 2. Results

### 2.1. α-AED Treatment Reduces the Viability of Murine Mammary Tumor 4T1 Cells and Human Breast Cancer Cells In Vitro

**Figure 1:**
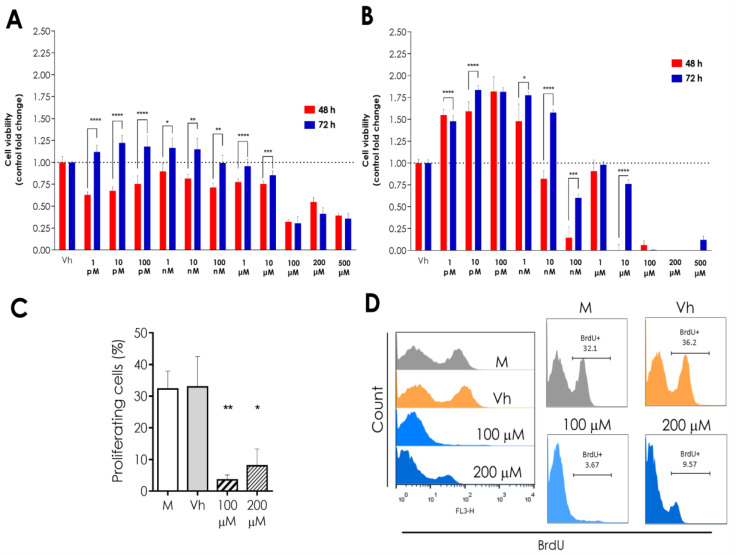
Effect of α-AED in 4T1 and HCC1937 cells. (**A**) 4T1 cells were exposed to vehicle (EtOH 0.2%) and α-AED 1 × 10^−12^ to 5 × 10^−4^ Molar (1 pM–500 μM) for 48 and 72 h. (**B**) Cell viability of HCC1937 triple-negative breast cancer cells was evaluated after 48 and 72 h of exposure to a vehicle (EtOH) or 1 × 10^−12^ to 5 × 10^−4^ Molar (1 pM–500 μM) of α-AED. Cell viability was measured through SRB assay and adjusted to the vehicle (Vh). Each bar represents the mean ± SEM. (**C**) Percentage of proliferating cells determined through BrdU incorporation. Graphs represent the mean ± SD of the data from four experiments (medium, *n* = 6; Vh, *n* = 6; AED 100 μM, *n* = 4; AED 100 μM, *n* = 4). (**D**) BrdU incorporation was determined in proliferative cells: offset histogram (left) and separated histograms of untreated 4T1 cells (M, medium), treated with vehicle (Vh), α-AED 100 μM and 200 μM. Statistical significance was calculated using Tukey’s multiple comparison test (*, *p* ≤ 0.05; **, *p* ≤ 0.01; ***, *p* ≤ 0.001; ****, *p* < 0.0001).

The proliferative effects of α-AED in 4T1 breast cancer cells were evaluated at different times (24, 48, and 72 h) and concentrations (1 pM–500 µM equivalent to 1 × 10^−12^ to 5 × 10^−4^ Molar). The results showed that this compound inhibited cell proliferation at 72 h, being more significant from the concentration of 1 × 10^−4^ Molar (Figure 1A). When comparing the proliferation rate evoked by α-AED at 1 × 10^−4^ Molar at different times (Figure 1A), we observed that the cell growth increased around 24% at 48 and 72 h after treatment as compared to 24 h of exposure. Although we found a biphasic effect at higher concentrations, it is important to highlight that cell proliferation was lower than at 24 h in contrast with the lower concentrations. We also assessed the effect of α-AED on the viability of the human triple-negative HCC1937 breast cancer cell line to compare the results found in the murine cell line with the effects of α-AED in a human breast cancer cell counterpart (Figure 1B). The proliferative effect of the HCC1937 cell line was also evaluated at 24, 48, and 72 h; however, the results of the significant effects were found in prolonged times of exposure. The data showed that after 72 h, the treatment of α-AED significantly increased the cell density of HCC1937 cells from concentrations between 1 × 10^−12^ and 1 × 10^−8^ Molar. Nevertheless, the cell concentration was drastically decreased at 0.1 µM, less than 50% of the control and the other concentrations tested (Figure 1B). 

To assess the α-AED antiproliferative effect, we measure BrdU incorporation in 4T1 proliferating cells treated with vehicle (EtOH 0.2%), α-AED 1 × 10^−4^ Molar (100 μM), and twice this concentration (200 μM or 2 × 10^−4^ Molar). Average BrdU positive cells were 9-fold lower when treated with α-AED 100 μM and 4-fold inferior with 200 μM (*p* ≤ 0.01 and *p* ≤ 0.05, respectively), compared with vehicle-treated cells (Figure 1C,D). 

Moreover, 4T1 and HCC1937 cell lines were exposed to titrated concentrations of α-AED for 24, 48, and 72 h (Supplementary Figures S1 and S2, respectively) to determine the dose of this steroid required to reduce 50% of cell proliferation as compared to cells treated with the vehicle (IC_50_). SRB assay was used to quantify the cell density by determining cellular protein content. IC_50_ for α-AED on the 4T1 cells tested is 100 μM and for HCC1937 is 100 nM.

A biphasic response is observed with the α-AED effect in 4T1 and HCC1937 cell lines (Figure 1A,B). The proliferation is increased in 4T1 cells treated for 72 h with lower concentrations (1 pM–10 nM of α-AED). At high doses, cell proliferation is suppressed (100 μM) and raised again (200 and 500 μM) (Figure 1A). This trend is similar in 4T1 cells treated for 48 h (Figure 1A). Meanwhile, in HCC1937 cells treated for 48 h, lower concentrations (1 pM–1 nM) proliferation is increased, then reduced at 10–100 nM, which is repeated with the increase with 1 μM of α-AED and the subsequent reduction. After 72 h, this trend is also similar (Figure 1A).

### 2.2. Intratumoral Injection with α-AED Reduced Tumor Size and Weight

**Figure 2:**
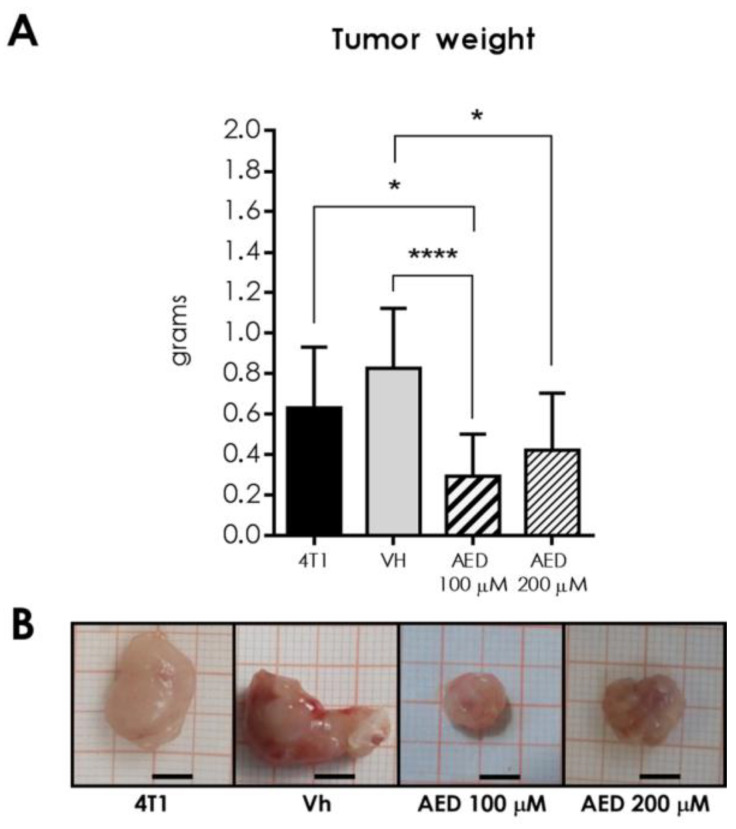
Effect of α-AED in 4T1 tumor weight in BALB/c mice. After 14 days of growth, tumors were injected with the vehicle—corn oil 40 μL—(Vh), α-AED 100 μM (1 × 10^−4^ Molar) or 200 μM (2 × 10^−4^ Molar), and excised after 28 days of tumor development. (**A**) Tumor weight from intact tumors (4T1), Vh, α-AED 100 μM, and α-AED 200 μM groups. (**B**) Representative images of tumors from all groups. Millimetric grid as a background; black bar = 0.5 cm. Graphs represent the mean ± SD of the data from three experiments. Statistical significance was calculated using the Kruskal–Wallis test (*, *p* ≤ 0.05; ****, *p* ≤ 0.0001).

After 28 days from 4T1 cell inoculation and 14 days after intratumoral injection, the mean weight of tumors treated with α-AED was smaller than the untreated (4T1) and the vehicle (Vh) injected tumors (Figure 2A,B). As observed in vitro (Figure 1), the average weight of tumors treated with α-AED 100 μM was smaller than those treated with twice this dose (200 μM) (Figure 2A,B). Tumor mass weight was 65% and 50% smaller than α-AED-treated groups (α-AED 100 = *p* ≤ 0.0001, α-AED 200 = *p* ≤ 0.05) compared with the Vh group.

### 2.3. α-AED In Situ Treatment Decreased Metastatic-like Macroscopic Lung Lesions

**Figure 3:**
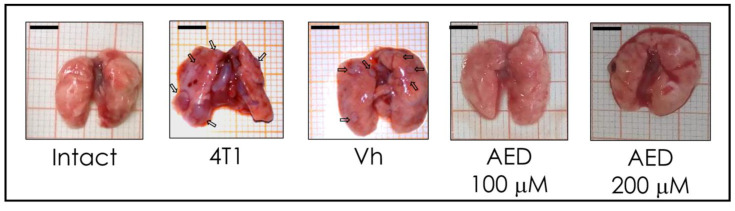
Effect of α-AED in metastatic lung lesions of 4T1 tumor mouse model. After 14 days of growth, tumors were injected with the vehicle—corn oil 40 μL—(Vh), α-AED 100 μM, and 200 μM. After 28 days of tumor development, the lungs of the mice were extracted and analyzed at the macroscopic level. Representative images of the lungs of the different experimental groups are displayed. Arrow refers to mestastatics stains.

Aside from assessing the effect of α-AED on tumor growth, we detected macro metastatic-like lesions on the lung surface. These macroscopic lesions were much more obvious in the non-treated tumors (4T1), as well as in vehicle-treated (Vh), while in the α-AED, there was an apparent decrease in the lesions (Figure 3).

### 2.4. α-AED In Situ Treatment Decreased Metastatic Microscopic Lung Lesions

**Figure 4:**
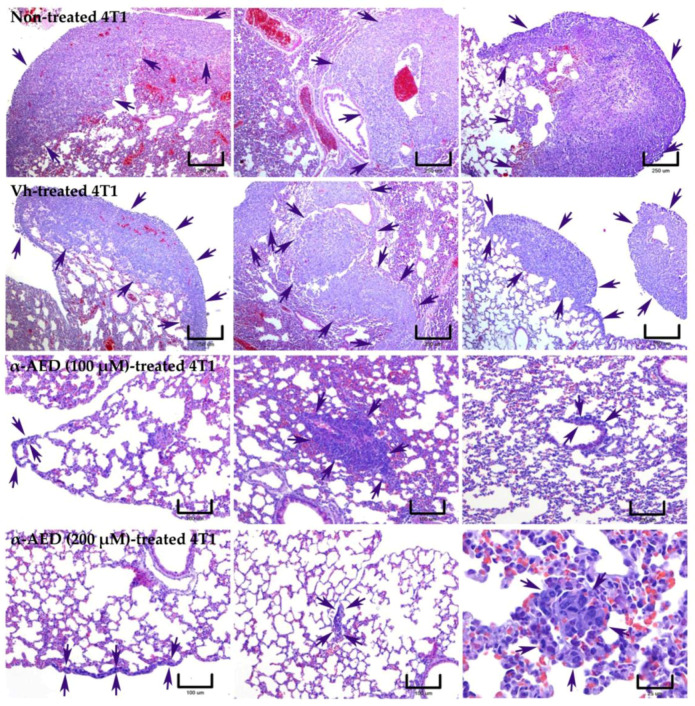
Effect of α-AED in histopathology and metastatic lung lesions of 4T1 tumor mouse model. The arrows point to and delimit the lung metastases in the 4 groups of mice. The images show that the lungs of mice with non-treated 4T1 and vehicle (Vh)-treated 4T1 tumors developed subpleural and parenchymal metastases notoriously more extensive than the lungs of mice with α-AED-treated 4T1 tumors, especially at the 200 μM dose. Note that inflammatory infiltration of the alveolar walls, the collapse of alveoli airspaces, and vascular congestion is most evident in the lungs of the first two groups of mice. To cover the microscopic field of the enormous metastases of these it was necessary to use the 4× objective (two upper panels; magnification bars = 250 µm). This explains the difference in magnification bars (two lower panels; magnification bars = 100, except the lower right = 25 µm). H&E stain.

Histopathological analysis of the lungs from mice with orthotopically implanted 4T1 tumors corroborated the necropsy findings. The size and number of subpleural and parenchymal lung metastases were higher in mice with non-treated 4T1 tumors and with vehicle-treated 4T1 tumors (Figure 4). It was necessary to observe with ×4 objective to photomicrographically record the greatest extent of metastases in these two groups of mice. In contrast, there was a reduction in the number and size of metastases in α-AED-treated mice, particularly in the lungs of mice with α-AED (200 μM)-treated tumors. In these last groups of mice, it was more convenient to make the photomicrographic record at greater magnifications (Figure 4). Additionally, other pulmonary histopathological differences between groups of mice were observed. The alveolar walls showed a diffuse and more intense inflammatory infiltrate in the vicinity of metastases, and even within these, in mice treated with 4T1 and Vh-treated 4T1 tumors, which caused the collapse of large areas of the pulmonary parenchyma. Conversely, in the pulmonary parenchyma from mice with α-AED (100 μM)-treated and α-AED (200 μM)-treated 4T1 tumors there appears to be a decrease in the diffuse inflammatory infiltrate, decreasing the presence of neutrophils but accompanied by an increase in mononuclear cells (Figure 4). Finally, vascular congestion of medium-sized blood vessels and capillaries was always much lower in the lungs of mice whose primary tumors were treated with α-AED.

### 2.5. Diminished Tumor Growth Is Associated with Minor Changes in the Tumor Microenvironment

**Figure 5:**
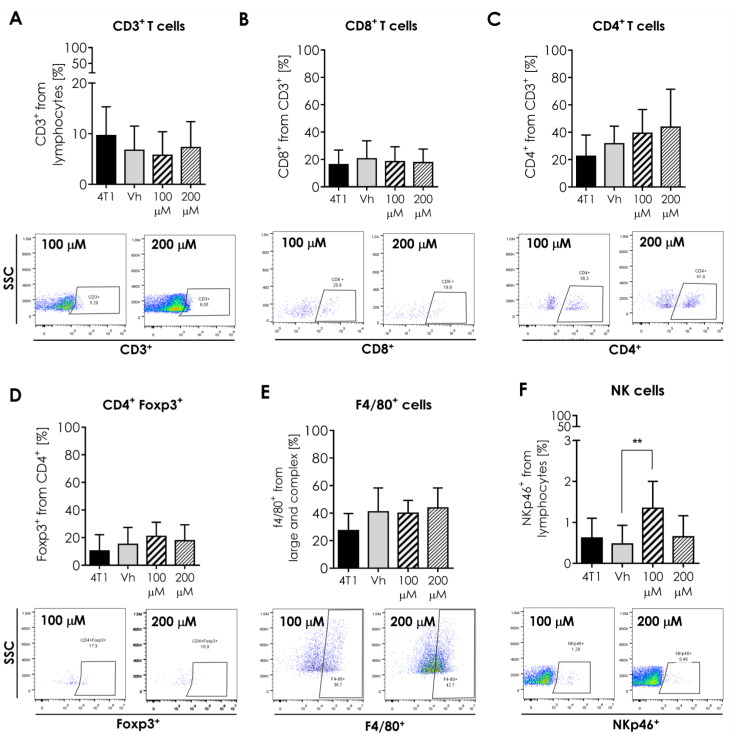
Effect of intratumoral α-AED treatment on immune cell infiltration in the 4T1 tumor model. Innate and adaptive immune cells in the tumor microenvironment. Determination of innate and adaptive immune subpopulations by flow cytometry within the tumor. (**A**) T CD3+ lymphocytes. (**B**) CD8+ T cytotoxic. (**C**) Helper CD4+ T lymphocytes. (**D**) CD4+/Foxp3+ lymphocytes. (**E**) F4/80+ macrophages. (**F**) NKp46+ cells. Graphs represent the mean ± SD of the data from three independent experiments. Representative dot plots of the cytometric analysis of the corresponding population of tumor cells (below). Gates from 10,000 cells were collected. Statistical significance was calculated using a Kruskal–Wallis test (** *p* ≤ 0.01).

Androstene steroids have been linked to immune response regulation; therefore, we determined some cell populations of innate (macrophages and NK cells) and adaptive (T and B lymphocytes) immune response within the tumor. Mean percentages of F4/80+ macrophages and T cells (CD3+, CD4+, CD8+, CD4+Foxp3+) intratumoral cells, remain without statistical difference (Figure 4). Meanwhile, the percentage of NK cells was higher (*p* ≤ 0.01) in tumors when treated with 100 μM (Figure 5). 

### 2.6. Humoral Response and B Cells in the Tumor

**Figure 6:**
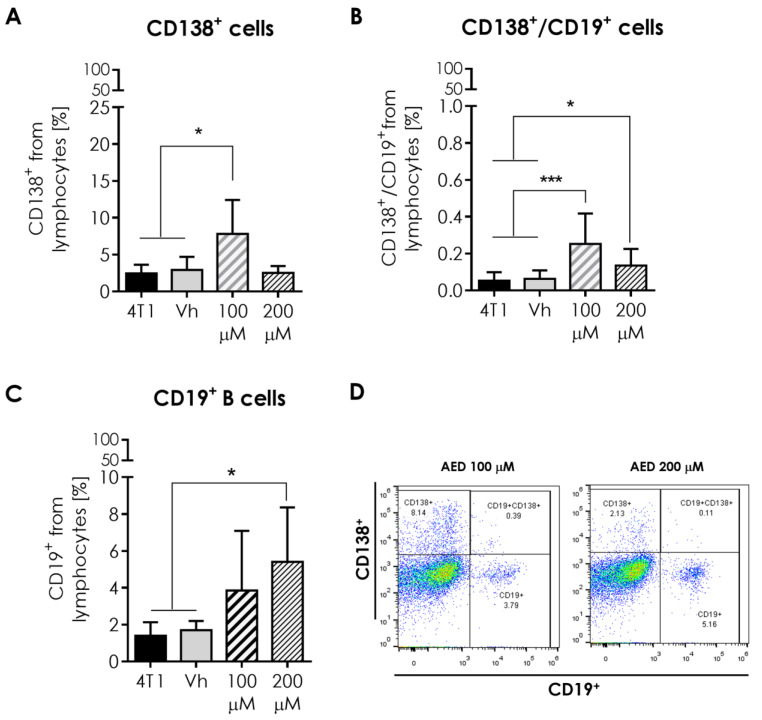
Effect of α-AED local injection on the humoral-related cell infiltration in the 4T1 tumor model. Humoral-related cell subpopulations by flow cytometry of the tumor. (**A**) Plasmatic cells (CD138+). (**B**) Plasmablasts (CD138+/CD19+). (**C**) B cells (CD19+). (**D**) Representative dot plots of the cytometric analysis of the corresponding population tumor cells. Graphs represent the mean ± SD of the data from three independent experiments. Gates from 10,000 cells were collected. Statistical significance was calculated using a Kruskal–Wallis test (*, *p* ≤ 0.05; ***, *p* ≤ 0.001).

Aside from the NK cell proportion rise, some changes were also noticed for the mean proportion of humoral-related cells inside the tumor (Figure 6A–D). The first of these changes was the high percentage (*p* ≤ 0.05) of plasmatic cells (CD138+) in the α-AED 100-μM-treated group (Figure 6A,B), and the second was the increase of B lymphocytes (CD19+) proportion (*p* ≤ 0.05) in α-AED 200 μM (Figure 6C,D). The third was associated to plasmablast cells (CD19+/CD138+) that had different proportions when treated with α-AED. Still, this population is very scarce inside 4T1 tumors (Figure 5B).

### 2.7. Microenvironmental VEGF Is Reduced in Tumors Treated with α-AED

**Figure 7:**
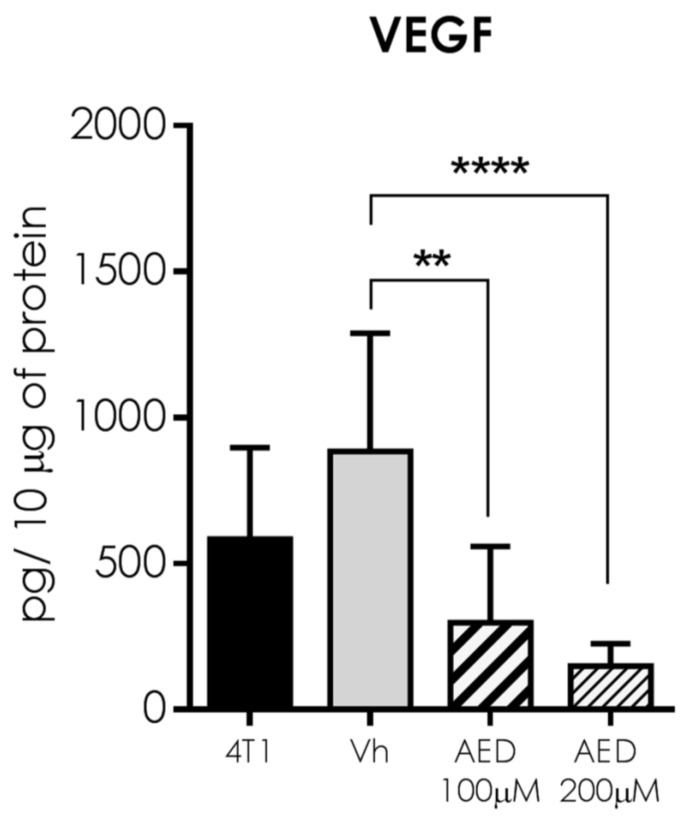
Analysis of the VEGF expression in the tumor microenvironment. Graph shows data from tumor proteins obtained from two independent experiments. Bars represent the mean ± SD of cytokine levels (pg/10 μg of tumor protein). Statistical significance was calculated using a Kruskal–Wallis test (** *p* ≤ 0.01; **** *p* ≤ 0.0001).

Because of the importance of tumor cell milieu communication to immune cell proportions, we search for another way by which α-AED could mediate the reduction of metastasis; thus, we determine tumor VEGF concentration. Notably, VEGF levels in α-AED-treated tumors were lower (100 μM = *p* ≤ 0.01, 200 μM = *p* ≤ 0.0001) than the Vh group (Figure 7). 

### 2.8. Tumor Cytokine Levels

**Figure 8:**
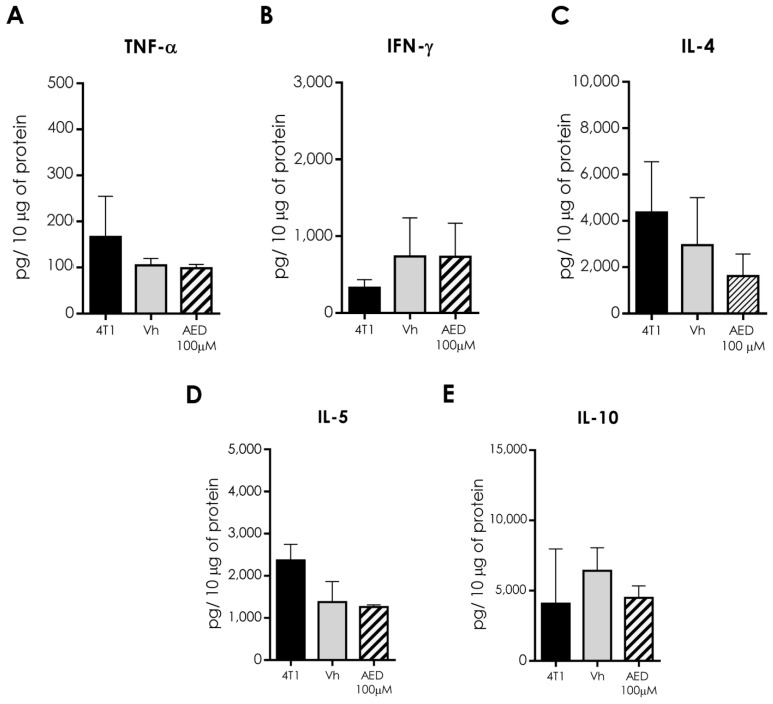
Expression of soluble factors in the tumor microenvironment. Analysis of (**A**,**B**) type 1 (TNF-α and IFN-γ), (**C**,**D**) type 2 (IL-4, IL-5), and (**E**) regulatory (IL-10) cytokines. Graphs indicate data of tumor proteins obtained from two independent experiments. Bars represent the mean ± SD of cytokine levels (pg/10 μg of tumor protein).

Since local treatment with α-AED 200 μM did not have the same effect on the reduction of tumor size compared with 100 μM intratumoral administration, we chose to measure some representative interleukins in the tumor of Vh and α-AED 100 μM only (Figure 8). We determined type 1 (TNF-α, IFN-γ Figure 8A,B), type 2 (IL-4 and IL-5 Figure 8C,D), and regulatory (IL-10 Figure 8E) cytokines. No changes were observed in these interleukins within the tumor (Figure 8A–E). 

### 2.9. Specific Humoral Response against 4T1 Cells Is Enhanced in Mice Treated with α-AED

**Figure 9:**
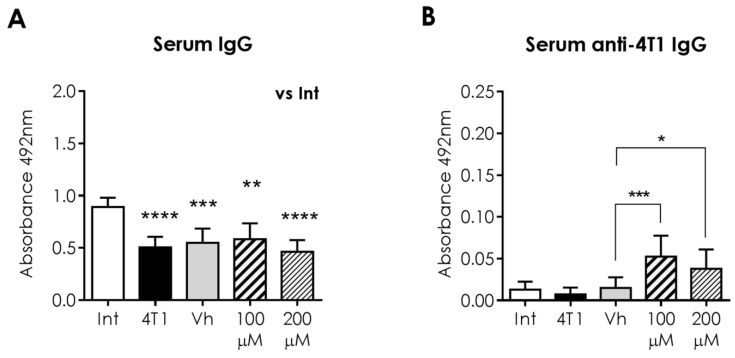
Systemic humoral response to intratumoral α-AED treatment in BALB/c mice. (**A**) Serum IgG levels (**B**) IgG anti-4T1 levels, in sera from intact (Int), tumor-bearing mice with no treatment (4T1), and sera from mice injected into the tumor with vehicle (corn oil 40 μL), α-AED 100 μM, or 200 μM. Graphs represent the mean ± SD of data from two experiments. Statistical significance was calculated using Kruskal–Wallis test (* *p* ≤ 0.05; ** *p* ≤ 0.01; *** *p* ≤ 0.001; **** *p* ≤ 0.0001).

In addition to this analysis, we obtained IgG and anti-4T1 IgG serum levels, of which unspecific levels of IgG (Figure 9A) in response to α-AED treatment remain with no statistical change. Nevertheless, anti-4T1-IgG from α-AED-treated mice were higher in serum from mice injected with 100 μM (*p* ≤ 0.001) and 200 μM of α-AED (*p* ≤ 0.05) (Figure 9B).

### 2.10. The Systemic Innate and Adaptive Immune Response Is Preserved in Tumor-Bearing Mice Treated with α-AED

**Figure 10:**
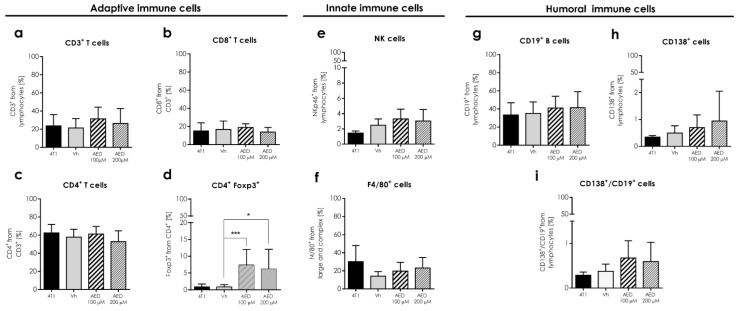
Effect of intratumoral α-AED treatment in the immune cell infiltration of the secondary lymphoid organ in the 4T1 tumor model. Determination of innate and adaptive immune subpopulations by flow cytometry in the spleen. (**a**) T lymphocytes CD3+. (**b**) CD8+ T cytotoxic lymphocytes. (**c**) CD4+ T helper lymphocytes. (**d**) CD4+/Foxp3+ T lymphocytes. (**e**) NKp46+ cells. (**f**) F4/80+ macrophages. (**g**) CD19+ B lymphocytes. (**h**) Plasmatic cells (CD138+). (**i**) Plasmablasts (CD138+/CD19+). Graphs represent the mean ± SD of the data from three independent experiments. Gates from 10,000 cells were collected. Statistical significance was calculated using a Kruskal–Wallis test (*, *p* ≤ 0.05; ***, *p* ≤ 0.001).

As part of the systemic humoral response analysis in tumor-bearing mice, we determined the percentage of B (CD19+), plasmatic (CD138+), and plasmacytoid cells (CD19+/CD138+) from the spleen. None of these three humoral-related cell proportions were different (Figure 10g–i). 

Because the injection of α-AED was performed inside the tumor, and we wanted to determine if this local treatment could reach a systemic level, we analyzed the effect of this local treatment on the systemic immune response in secondary lymphoid organs to assess whether these modifications in splenic cell proportions were different in treated and untreated mice (Figure 10a–i). Innate and adaptive cells obtained from the spleen of mice treated with α-AED conserved their proportions when compared to the Vh group, except for CD4+/Foxp3+ T cells. The ratio of this regulatory population was augmented in the spleen of tumor-bearing mice treated with α-AED 100 μM (*p* ≤ 0.001) and with α-AED 200 μM (*p* ≤ 0.005) (Figure 10d).

### 2.11. The Systemic Innate and Adaptive Immune Response Is Preserved in Tumor-Bearing Mice Treated with α-AED

**Figure 11:**
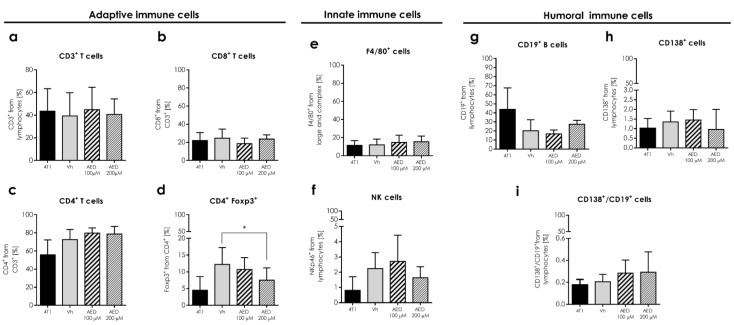
Effect of intratumoral α-AED treatment in the immune cell infiltration of secondary lymphoid organs in the 4T1 tumor model. Determination of innate and adaptive immune subpopulations by flow cytometry in lymph nodes. (**a**) T lymphocytes CD3+. (**b**) CD8+ T cytotoxic lymphocytes. (**c**) CD4+ T helper lymphocytes. (**d**) CD4+/Foxp3+ T lymphocytes. (**e**) F4/80+ macrophages. (**f**) NKp46+ cells. (**g**) CD19+ B lymphocytes. (**h**) Plasmatic cells (CD138+). (**i**) Plasmablasts (CD138+/CD19+). Graphs represent the mean ± SD of the data from three independent experiments. Gates from 10,000 cells were collected. Statistical significance was calculated using a Kruskal–Wallis test (*, *p* ≤ 0.05).

It is interesting to note that the same pattern found in the spleen was found in the closer lymphoid organ to the tumors, the inguinal lymphatic nodes (Figure 11a–i), except for Treg cells (CD4+/Foxp3+) (Figure 11d). However, this reduction of inguinal lymph node regulatory cells proportion was only significant when tumors were treated with α-AED 200 μM (*p* ≤ 0.005) (Figure 11d).

## 3. Discussion

The steroid α-AED has been widely tested as a proliferation inhibitor in different cancer cell lines, and its inhibitory concentrations vary according to the type of cell line. The inhibitory effect of α-AED has been proved in breast cancer cell lines in vitro. Examples include breast adenocarcinoma (MCF-7, MDA-MB231) and mammary ductal carcinoma (T-47D and TTU-1) with a lethal dose (LD50) established between 8 and 15 μM [6]. It is important to mention that, even though the LD50 found in our results it is high, it does not exceed that found in human cancer cell lines. Thus, we are on the safe side in that regard.

The biphasic proliferative effect induced by the treatment of α-AED that was observed in our results is similar to that found in other cell models, where higher concentrations of the 17β-AED and 17α-AED steroid compounds induced cell proliferation in higher concentrations, unlike the inhibitory cell effect shown at lower concentrations [16]. It is important to highlight that, as in previous reports, our results did not significantly increase the proliferative rate. Moreover, the biphasic effect of different hormones in the proliferation of cancer cells has been widely reported [17,18]. In addition, a biphasic proliferative response has been observed associated with the α-AED effect. First, the proliferation is increased in 4T1 cells treated with lower concentrations (1 pM–10 nM of α-AED). This effect has also been observed in murine macrophage-like cell line RAW 264.7 (concentration lower than 6.25 nM) cultured for 48 h and in human breast ductal carcinoma cells ZR75-1, as well as MDA-MB231 breast tumor cells (lower than 12.5 nM) cultured during six days [8]. Second, at high doses, cell proliferation is suppressed (100 µM) and raised again (200 and 500 µM). This biphasic phenomenon is associated with androgens and reported in the LNCaP human prostatic cancer cell line treated with dihydrotestosterone (DHT). Therefore, it is proposed that high physiologic doses could be a tumor treatment [19,20].

In agreement with the results of Graf et al., we also observed that the triple-negative breast cancer cell line HCC1937 displayed low sensitivity to the inhibitory cell viability effect of the α-AED. The authors evaluated the effects of α-AED in the MDA-MB-231 triple-negative breast cancer cell line, showing its best result at 10–15 μM when compared with the MCF-7 cell line (IC_50_ around 8.0 µM). The antiproliferative effects modulate eukaryotic initiation factor 2 (eIF2) more than ERα status. Regarding the mechanism of action of α-AED, a weakness of this work is that different mechanisms by which the compound acts in triple-negative breast cancer cells have yet to be elucidated in future studies. Therefore, we extended the insights evoked previously by dehydroepiandrosterone in other breast cancer cells similar to α-AED, where a reduction in cell proliferation has been achieved with high doses of the adrenal hormone dehydroepiandrosterone [4,6,21]. Besides, we hypothesize that these findings also open the study for searching different cell targets with alternative techniques that allow exploring what mechanisms are due to the sensitivity of cancer cells to α-AED. Our research group has recently reported that although it was not declared, a direct proliferative effect of different estrogenic compounds in human triple-negative breast cancer cells, several crucial molecular factors involved in epithelial transition or hypoxia processes, among others, were modulated after their exposure [22].

In line with the reduction of 4T1 cell density treated with α-AED 100 μM and 200 μM concentrations (*p* ≤ 0.01 and *p* ≤ 0.05) (Figure 1A,B), we assessed if this effect was associated with the inhibition of the proliferation through the BrdU incorporation in proliferating cells. After 72 h of treatment with 100 μM and 200 μM, we observed a dramatic reduction in the percentage of proliferating cells BrdU+ (*p* ≤ 0.01 and *p* ≤ 0.05, respectively) (Figure 1C,D). These results suggest that the reduction in cell density detected through SRB assay was associated with decreased proliferation of 4T1cells treated with α-AED [7].

After assessing the size reduction of tumors treated with 100 μM and 200 μM of α-AED, we determined the effect of intratumoral treatment on immune cell infiltration in the 4T1 tumor model. According to Loria, 2002, immune regulation is associated with β-AED exposure, which is chemically identical to α-AED; meanwhile, the anti-tumoral effect is linked with the α-AED treatment [8,23]. However, because of the structural similarities between androstenediol β and α, and the lack of information associated with the immune tumor cell microenvironment, we search for some immune components of this milieu to determine if α-AED intratumoral treatment exerts local immune changes.

Moreover, cancer is a complex disease that involves tumor development and cell metastasis from the tumor to other distant tissues. Primary tumor growth rarely causes the death of its carrier and is the metastatic disease that causes the vast majority (about 90%) of cancer-related deaths [24]. In this sense, we observed that lungs from 4T1 group mice exhibited metastatic-like lesions on the lung surface. These macroscopic metastases disappeared when tumors were treated with α-AED (100 μM and 200 μM) (Figure 3). For instance, NK cell proportion was augmented within the tumors treated with α-AED 100 μM (Figure 4). NK cells are known as cytotoxic cells of the innate response and in tumors, are principal effectors of the cancer immunoediting by recognizing and destroying tumor cells directly through the exocytosis of granules with perforin and granzyme, apoptosis mediated by different death receptors (FasL, TRAIL, and TNF-α), and IFN-γ secretion [12,25,26]. Another way NK cells exert their cytotoxic effect in solid tumors is by the antibody-dependent cell-mediated cytotoxicity activated by antibodies linked to target cells [27].

With these findings in the present study, we also search for humoral-response components in the tumor milieu as follows. In treated tumors, the percentages of plasmatic CD138+ and plasmablast cells CD19+ +/CD138+ were higher in tumors injected with 100 μM of α-AED (*p* ≤ 0.05 and *p* ≤ 0.001, respectively) (Figure 5A,B). Furthermore, the tumors injected with 200 μM of α-AED contained an elevated proportion of plasmablast and B cells CD19+ (*p* ≤ 0.05) (Figure 5B,C). To our knowledge, there is no report about the effect of α-AED on the humoral-related response. Thus, these findings are the beginning of a further investigation. Moreover, the presence of naïve or mature B cells within different types of breast tumors is related to a positive prognostic effect in 54 cohorts [28]. Furthermore, B (CD19+) lymphocytes are associated with a favorable prognosis in the overall survival of patients with tongue squamous cell carcinoma [29]. In a similar case, elevated plasma cell density is linked to a longer time to relapse in triple-negative breast cancer tumors (TNBC) [30]. Consequently, according to this knowledge and the increased proportions of B and plasmatic cells in the milieu of α-AED-treated tumors, we determined the systemic humoral response of these tumor-bearing mice. Neither of the populations’ changes observed in the tumor was detected in the spleen or PLN (Figure 6)

In addition to immune cell proportions, we search for complementary ways by which α-AED mediates the reduction of the tumor size. Thus, we determined tumor soluble factors that include angiogenic (VEGF), type 1 response (TNF-α, IFN-γ), type 2 response (IL-4, IL-5), and regulatory (IL-10) soluble factors. Notably, VEGF levels in α-AED-treated tumors were lower (100 μM = *p* ≤ 0.01) than in the Vh group (Figure 7). As previously mentioned, VEGF is related to vessel growth, nutrient supply, and tumor cell dispersion to other tissues; thus, its decrease within the tumor may reduce tumor growth and metastasis [13]. The diminished concentration of VEGF level in α-AED 100-μM-treated tumors may be associated with a direct production inhibition of the tumor milieu cells or linked to other microenvironmental factors. The VEGF expression is modified by hypoxia, free radicals, pH imbalance, and nutrient deficiency, which increase when the tumor grows. Therefore, the inhibition of the proliferation of the 4T1 cells mediated by α-AED could reduce these pro-angiogenic factors within the treated tumors and, consequently, the VEGF production [31]. Further studies to unveil these mechanisms are needed. Since local treatment with α-AED 200 μM did not have the same effect on the reduction of tumor size compared with the intratumoral administration of α-AED 100 μM, we chose to exclude this group from this determination (Figure 8). No type 1, type 2, and regulatory response interleukins were different in α-AED-injected tumors (Figure 6 and Figure 8E).

Intratumor drug delivery is not a common practice in breast cancer patients, with intravenous systemic chemotherapy being the most common administration route. However, only a tiny fraction of the anticancer drug reaches the tumor; therefore, high doses of the medicament must be administrated, increasing the undesirable side effects of chemotherapeutic compounds that affect healthy tissues [32]. A leading objective in cancer research is to avoid or reduce the toxicity of conventional cytotoxic treatments. A promising method is the local delivery of the anticancer drug [33], which may be used as a neoadjuvant or combinatory therapy before tumor removal. For that reason, different studies on diverse cancer types have focused on other intratumoral alternatives that have been proposed and applied in some cases in clinical and palliative cancer therapy [34,35], highlighting the translational impact of this work. Thus, in this study, we injected the antiproliferative steroid α-AED into the tumor. Besides its local immunological changes, we looked for its systemic immune effect in secondary lymphoid organs, such as the spleen and PLN. Following the previous findings, the reduction in tumor size accompanied by changes in humoral-related responses within the tumor treated with α-AED, suggests these cells’ role in tumor growth control. This could be explained because the B lymphocytes promote T-cell responses in the tumor microenvironment by producing cytokines and chemokines; and that they also differentiate into antibody-producing plasmatic cells [28]. Specific antibodies may recognize tumor-associated antigens and act directly on target proteins or, through their Fc receptor, trigger antibody-dependent cellular cytotoxicity (ADCC) and improve antigen presentation and activation of T cells [28]. Thus, to improve our understanding of humoral-related mechanisms in the 4T1 tumor mice model treated with α-AED, we determined the unspecific IgG and anti-4T1 IgG levels in mice serum. As well as B, plasmatic, and plasmablasts cells, we found no changes in unspecific antibodies (IgG). In contrast, the 4T1-specific IgG levels were elevated in mice treated with 100 μM and 200 μM of α-AED (Figure 6).

Furthermore, cancer immunotherapy strategies may activate the immune system, but they can also lead it into supraphysiological levels with a subsequent risk of increasing immune-related adverse events. That is why targeted or localized drug delivery should be a major goal of chemotherapy. In general, intratumoral immunotherapies aim to initiate local recruitment of immune cells into the tumor microenvironment and subsequently prime T cells for a systemic polyclonal antitumor response and enhance this response, however, they can also directly attack the transformed cells, thus the natural immune control can be restored, and tumor growth contained. The intratumoral strategy offers enhanced regional efficacy and reduced systemic toxicity by enabling high bioavailability of the agent at the injected tumor sites while limiting systemic exposure and possibly systemic toxicity. Despite there being some information available for several types of cancer, the use of these new strategies in breast cancer have been poorly explored and the translation of proved strategies, as well as the development of new specific ones, remains urgent. Our results demonstrated that with a single local intratumoral injected dose of AED, tumors were reduced significatively as well as lung metastasis. Nevertheless, more studies are needed to determine the number of doses needed for a complete tumor growth inhibition and 100% metastasis inhibition as well. 

Intratumoral immunotherapy treatments definitely reduce toxic effects and enhance the aimed responses. One of the main concerns about intratumoral injections was the possibility to “open the door” to metastasis; nevertheless, the evidence points to the fact that our intratumoral treatment avoids metastasis. The contact time between the therapy and the tumor cells seems to be critical in order to trigger specific immunity against tumors. Effectiveness of single intratumoral injections is related to the tumor size, the number, and sites of given doses. That is why a homogenized distribution and long-lasting delivery must be achieved in further research in order to increase the success of the intratumoral treatments. In order to achieve this goal, the combination of the administration technologies with the delivery strategies will be critical. It will also be desirable to personalize the treatments as much as possible, since not all patients respond to the same strategies, even if they bear the same type of breast cancer. This is the beginning of the translational study, and thus our results are very exciting in the context of the clinical presentation of the disease. Despite the significant advances in medicine, breast cancer remains one of the leading causes of death, and the number of breast cancer cases is still increasing worldwide. Moreover, it would be important to evaluate the combination of the α-AED-based therapy with other kinds of already available ones, or new promising drugs, to try out to interrupt the co-evolution of the immune system and tumor cells. Thus, the present contribution holds the basis to use α-AED alone or as a combined therapy with those already in use or in clinical tests to decrease the burden of breast cancer. 

## 4. Materials and Methods

### 4.1. Reagents

The α-AED was supplied by Dr. Roger Loria (Virginia Commonwealth University). All additional reagents used were acquired as described below. All procedures were performed at the Instituto de Investigaciones Biomédicas (IIB) from the Universidad Nacional Autónoma de México (UNAM).

### 4.2. Ethics Statement

The Institutional Care and Animal Use Committee (CICUAL) reviewed and approved the animal study. All the experimental procedures and animal studies were performed within the standards established by CICUAL (permit number 2017-208), under Mexican regulation (NOM-062-ZOO-1999), and with the Guide for the Care and Use of Laboratory Animals of the National Institute of Health (NIH) of the United States of America. Animal procedures were performed at the IIB, UNAM, in the Biological Models Unit (Unidad de Modelos Biológicos, UMB, IIB, UNAM, Mexico, City).

### 4.3. Animals

Female 8-week-old BALB/c AnN mice (MGI Cat# 5654849, RRID: MGI:5654849) were obtained from Envigo México (Facultad de Química, UNAM, México). The animals were housed 4–5 per cage with 12 h of alternating light at 22 °C. Water and food (Envigo LabDiet 5015-Cat# 0001328 Purina, St. Louis, MO, USA) were delivered ad libitum in sterile conditions.

### 4.4. Cell Culture

The 4T1 murine mammary carcinoma cell line and the HCC1937 and a human breast cancer cell line (ATCC Cat# CRL-2539, RRID: CVCL_0125) were cultured in RMPI 1640 medium (Sigma, St. Louis, MO, USA) supplemented with 10% fetal bovine serum (FBS) for murine cells and 5% FBS for the human cell line (ByProducts, Guadalajara, Mexico), 1.0 mM sodium pyruvate, 100 units/mL penicillin, and 100 mg/mL streptomycin. 4T1 cells were maintained at 37 °C, with 95% humidity and 5% CO_2_ atmosphere. 

### 4.5. Cell Viability Assay and IC50 Determination

To determine the dose of this steroid required to reduce 50% of the cell proliferation compared to cells treated with the vehicle (IC50), the SRB assay was used to quantify the cell density by determining cellular protein content. For the Sulforhodamine B (SRB) assay (Sigma, St. Louis, MO, USA), 4T1 and the triple-negative breast cancer cell line and HCC1937 cells were seeded in 96-well culture plates (1 × 10^3^–2 × 10^3^ cells per well) in media supplemented with 5 or 10% heat-inactivated and charcoal treated-FBS, 100 units/mL penicillin plus 100 μg/mL streptomycin, and maintained with a 5% atmosphere of CO_2_ at 37 °C and 95% humidity. Twenty-four hours after seeding, cells were treated once with vehicle (0.1% *v*/*v* EtOH) or with increasing concentrations of α-AED and incubated for 72 h. Next, cell viability was obtained using an SRB assay that determines cell density by measuring protein content, performed according to [36], with modifications. In brief, cells were fixed with 100 μL of cold trichloroacetic acid (10%) for 1 h, washed three times with water, and air-dried overnight. Afterwards, cells were stained with 100 μL of SRB 0.057% in 1% acetic acid for 30 min, washed with 150 μL of acetic acid (1%), and air-dried for 2 h. Next, 100 μL of 10 mM Tris base solution (pH 10) was aggregated to solubilize the protein-bound dye, and the plates were agitated for 30 min. Plates were read at 492 nm in a Stat Fax 4200 microplate reader (Awareness Technology, Costa Mesa, CA, USA). Data were normalized to run between 0 and 100% and 0 and 1 as follows: Cell viability = optical density (OD) 492 in treatment wells/OD492 in control wells, respectively. Experiments were performed in triplicate on three different occasions, organized as an XY tab, and charted as a dose-response curve fitted to determine the IC50; calculated with Prism 6 software (GraphPad Software Inc., San Diego, CA, USA).

### 4.6. Cell Proliferation Assay

To quantify 4T1 cell proliferation, we determined the bromodeoxyuridine (BrdU) incorporation during DNA synthesis. This assay was performed in cells maintained in the same culture conditions as those for the SRB assay and treated with vehicle, 100 μM, and 200 μM, for 72 h. Procedures were performed according to the manufacturer’s protocol (BD Apoptosis, DNA Damage and Cell Proliferation Kit, Cat 15821759, Thermo Fischer Scientific, Waltham, MA, USA) with PerCP-Cy5.5 Mouse Anti-BrdU. Briefly, cells were incubated with BrdU at a final concentration of 10 μM in cell culture medium (10^6^ cells/mL). The treated cells were then incubated for 2 h. Then, 4T1 cells were fixed and permeabilized in sequence with BD Cytofix/Cytoperm Buffer, BD, and BD Cytofix/Cytoperm Buffer. Finally, cells were treated with DNase before incubation with the anti-BrdU-FITC antibody. Cell analysis was performed with BD FACSCalibur^TM^ (BD Biosciences, Switzerland Sàrl) flow cytometer and the data were analyzed with FlowJo software (Treestar Inc., Switzerland Sàrl). Compensation was assessed in BD FACSCalibur^TM^ and FlowJo software 3 with unstained samples.

### 4.7. Orthotopic Tumor Cell Induction

After a second subculture at 80% confluency, 4T1 cells were harvested and suspended in sterile 0.9% NaCl solution at a concentration of 250,000 cells/mL. They were conserved in ice until injection into the mice. For the mammary tumor induction, mice were anesthetized with sevoflurane 5% (Abbot, Mexico City, Mexico), and the abdominal area was cleaned with EtOH 70%. In the fat pad under the second last right nipple, 1 × 10^4^ 4T1 cells were injected subcutaneously. Mice recovery was supervised.

### 4.8. Tumor Model and α-AED Treatment

To test the intratumoral effect of α-AED on tumor growth, female mice were randomized into the following experimental groups: (1) Intact group of animals (*n* = 11); (2) 4T1 group of animals with untreated tumors (*n* = 12); (3) Vh group with tumors injected with 40 μL of corn oil vehicle (*n* = 14); (4) α-AED 100 μM group of mice with intratumoral injection of 580 ng of α-AED in 40 µL of corn oil (*n* = 15), and (5) α-AED 200 μM group of animals injected with 1160 ng of α-AED in 40 μL of corn oil into the tumor (*n* = 12). Tumor growth was observed for 28 days. Finally, the tumor weight was obtained at the moment of the euthanasia on day 28 post-inoculation.

### 4.9. Flow Cytometry

General tumors were excised and minced with a scalpel. The left and right peripheral (inguinal) lymph nodes (PLNs) and the spleen were excised and mechanically disaggregated through a 50 μm nylon mesh with PBS. After the PBS wash, the cells of the lymph nodes were resuspended in FACS buffer (PBS, 2% FBS, 0.02% NaN3). Splenic erythrocytes were lysed with ACK buffer (150 mM NH4Cl, 10 mM KHCO3, 0.1 mM Na2 EDTA, pH 7.3) for 10 min, washed with PBS, and resuspended in FACS buffer. The minced tumors were incubated in a digestion medium (RPMI 1640, 10 U/mL DNase, Roche, Mannheim, Germany; 0.5 mg/mL type IV Collagenase, Sigma, St. Louis, MO, USA) for 20 min. To stop the tumor digestion, 50 μL FBS was added, and the mechanical disruption in a 50 μm nylon mesh was performed. After the PBS wash, the cells were resuspended in FACS buffer. Approximately 1 × 10^6^ cells were incubated (20 min at 4 °C) with anti-CD16/CD32 (TruStain, Cat# 101319, Clone 93, RRID:AB_1574973, BioLegend, San Diego, CA, USA) and washed. Then, they were stained with the following panels. For T lymphocyte: AlexaFluor 488-conjugated anti-CD3ε (Cat# 100321, Clone 145-2C11, RRID:AB_389301) 1:100, PE-conjugated anti-CD4 (Cat# 100407, Clone GK1.5, RRID:AB_2075573) 1:300, PerCP-conjugated anti-CD8 (Cat# 100732, Clone 53–6.7, RRID:AB_893423) 1:100, and AlexaFluor 647-conjugated anti-Foxp3 (Cat# 320013, Clone 150D, RRID:AB_439750) 1:100. For macrophage and NK: AlexaFluor 647-conjugated anti-F4/80 (Cat# 123122, Clone BM8, RRID:AB_893492) and PE-conjugated anti-NKp46 (Cat# 137604, Clone 29A1.4, RRID:AB_2235755). To B lymphocyte: PE-conjugated anti-CD19 (Cat# 115507, Clone 6D5, RRID:AB_313642), 1:200, and plasmatic cells: Brilliant Violet 421TM anti-mouse CD138 (Syndecan-1) (Cat# 142507, Clone 281-2, RRID: AB_2565621). Antibodies from BioLegend, San Diego, CA, USA, and the Foxp3/Transcription Factor Staining Buffer kit (Cat# TNB-0607-KIT, Tonbo Biosciences, San Diego, CA, USA) were used for intracellular Foxp3 staining, according to the manufacturer’s protocol.

Cell analysis was performed with the BD FACSCalibur^TM^ (BD Biosciences) flow cytometer. The data were analyzed with FlowJo software (Treestar Inc.). Compensation was assessed in BD FACSCalibur^TM^ and FlowJo software with unstained samples, single stain controls, and FMO for Foxp3+ (CD3+/CD4+).

### 4.10. Cytokine Determination

The tumors from the mice were stored in TRIzol^TM^ reagent (Cat# 15596026, Invitrogen, Waltham, MA, USA) at −70 °C until use. Protein isolation was performed according to the procedural guidelines for TRIzol^TM^ reagent use. Protein quantification was performed with a NanoDrop 1000 spectrophotometer (Thermo Scientific, Waltham, MA, USA). To determine cytokine tissue levels, 10 µg of protein was used.

Tumor cytokines were measured with ABTS ELISA kits (PeproTech, Waltham, MA, USA) and the antibodies: TNF-α (Cat# 500-P64bt, RRID:AB_147984), IFN-γ (Cat# 500-P119bt, RRID:AB_148087), IL-4 (Cat# 500-P54bt, RRID:AB_147636), IL-5 (Cat#500-P55), and IL-10 (Cat# 500-P60, RRID:AB_147978). The same unconjugated antibodies were used for cytokine capture, according to the manufacturer´s instructions. Briefly, coated plates (96-well plate, MaxiSorp Nunc Cat# NNC#442404) with 50 µL (2 µg/mL) of different antibodies were incubated overnight. After 3 three washes (wash buffer, PeproTech), the plates were blocked (block buffer: PeproTech) and washed again. Samples were added by duplicate as follows: 50 µL of sera (1:2 dilution) or tissue protein (10 µg) (in diluent solution, PeproTech), incubated at 4 °C for 2 h and washed three times. An enzyme-substrate reaction was developed with ABTS liquid substrate (PeproTech). The plates were read at 405 nm wavelength with a wavelength correction set at 650 nm at different time points in a Stat Fax 4200 microplate reader (Awareness Technology). All solutions were from the ABTS ELISA buffer kit (Cat# 900-K00).

### 4.11. VEGF Quantification

The VEGF concentrations were calculated by interpolation from a standard curve (0.001–1 ng/mL) performed with the mouse VEGF protein, VEGF mBA-165 (Cat# sc-4571, Santa Cruz Biotechnology, Santa Cruz, CA USA). Tumor protein was obtained and quantified through the same protocol implemented for cytokine determination.

The polystyrene wells (96-well plate, MaxiSorp Nunc Cat# NNC#442404) were coated with 50 µL of tumor protein (10 µg) or with the different concentrations of the standard curve, all diluted in bicarbonate buffer (pH 9.6), coated per duplicate, and incubated at 4 °C overnight. The plate was washed and blocked with 200 µL of PBS/bovine serum albumin (BSA) 1%/Tween 20 0.05% for 1 h at 4 °C and washed again. Furthermore, 50 µL of anti-VEGF/C-1 antibody (Cat# sc-7269, RRID:AB_628430, Santa Cruz Biotechnology) in a 1:200 dilution was added and incubated for 1 h at 4 °C. After washing, 50 µL of m-IgGκ/BP-HRP (Cat# sc-516102, RRID:AB_2687626, Santa Cruz Biotechnology) (1:400) was added and maintained for 2 h at room temperature. An enzyme-substrate reaction was developed with 50 µL of substrate solution and stopped after 15 min with 50 µL 2N sulfuric acid. The plates were read at a wavelength of 492 nm in a Stat Fax 4200 microplate reader (Awareness Technology). Cytokine and VEGF concentrations were calculated by interpolation from a standard curve.

### 4.12. IgG Detection

Polystyrene wells (96-well plate, MaxiSorp Nunc Cat# NNC#442404) were coated with 50 µL of mice sera/bicarbonate buffer (pH 9.6) (1:1000) or with 1 µg/1 mL of 4T1 crude extract, overnight at 4 °C. The plate was washed three times with PBS/Tween 20 0.05% and blocked with 200 µL of 3% BSA and washing solution for 30 min at room temperature. The plate was washed as mentioned. For the anti-4T1 IgG determination, plates were incubated with mice sera (1:200) for 2 h at room temperature and washed. In both cases, 50 µL of peroxidase goat anti-mouse IgG (Jackson, PA, USA RRID:AB_2338511) at 1:10,000 dilution was added over 90 min at room temperature. An enzyme-substrate reaction was developed by adding 50 µL of freshly prepared substrate solution (0.05% o-phenylenediamine/0.01% H_2_O_2_/0.1 M sodium citrate/0.1 M citric acid) and stopped with 50 µL 2N sulfuric acid after 10 min. The plate was read at a wavelength of 492 nm in a Stat Fax 4200 microplate reader (Awareness Technology). Cytokine, VEGF, and antibody determination were performed after proper ELISA standardization.

### 4.13. Histological Analysis of Lungs

According to the Mexican Official Guide, all mice were sacrificed by isoflurane overdoses. To prevent alveolar collapse, whole lungs were fixed through intratracheal perfusion with 500 µL of 4% paraformaldehyde diluted in isotonic saline solution. After thoracotomy, lungs were dissected and then submerged in the same fixative solution for a minimum of 24 h. Furthermore, the lungs were rinsed in tap water, dehydrated through ascending ethanol grades, cleared in xylene, and embedded in paraffin with an orientation to obtain transversal sections from both lungs. Sixteen lung histological sections from each group of mice (4 μm thick and separated from each other by 100 μm) were stained with hematoxylin-eosin and analyzed using a BX50 Olympus microscope equipped with a digital camera and Infinity Analyze software, v6.3.0.

### 4.14. Statistical Analysis

Data were charted as mean ± SD. A non-parametrical Kruskall–Wallis test and Tukey test for multiple comparisons were applied. The differences were considered significant when *p* ≤ 0.05. All the analyses were calculated with Prism 6 software (GraphPad Software Inc.).

## 5. Conclusions

In summary, we have developed an intratumor (in situ) treatment with α-AED. This steroid exerts an antiproliferative effect on human and murine cancer cells and modulates the immune response in a mouse breast cancer model. Using this strategy, we showed that local delivery of α-AED might enhance different therapies and, through the downregulation of VEGF secretion, also reduce metastasis. Furthermore, compared to other cancer treatments, α-AED is an affordable option that lacks the undesirable toxic effects of chemotherapeutic drugs. Our results highlight the great potential of α-AED for mediating antitumor activity and metastasis reduction will surely need to be proven as a neoadjuvant or adjuvant treatment in animals and humans. Therefore, our results offer a significant bridge between basic and preclinical medicine toward translational studies as an urgent demand for therapeutic options.

## Data Availability

The datasets generated and analyzed during the current study are included in the present manuscript. Furthermore, they are available from the corresponding author on request.

## Supplementary Materials

The following supporting information can be downloaded at: https://www.mdpi.com/article/10.3390/ijms231911944/s1.
