# Supplementary material for: Intratumoral Treatment with 5-Androstene-3β, 17α-Diol Reduces Tumor Size and Lung Metastasis in a Triple-Negative Experimental Model of Breast Cancer
Source: Int J Mol Sci. 2022 Oct 8;23(19):11944. doi: 10.3390/ijms231911944 (PMC9570136; doi:10.3390/ijms231911944)
Supplement: Supplementary file 1 [file ijms-23-11944-s001.zip › ijms-1775130-supplementary.pdf]

Supplementary Materials:

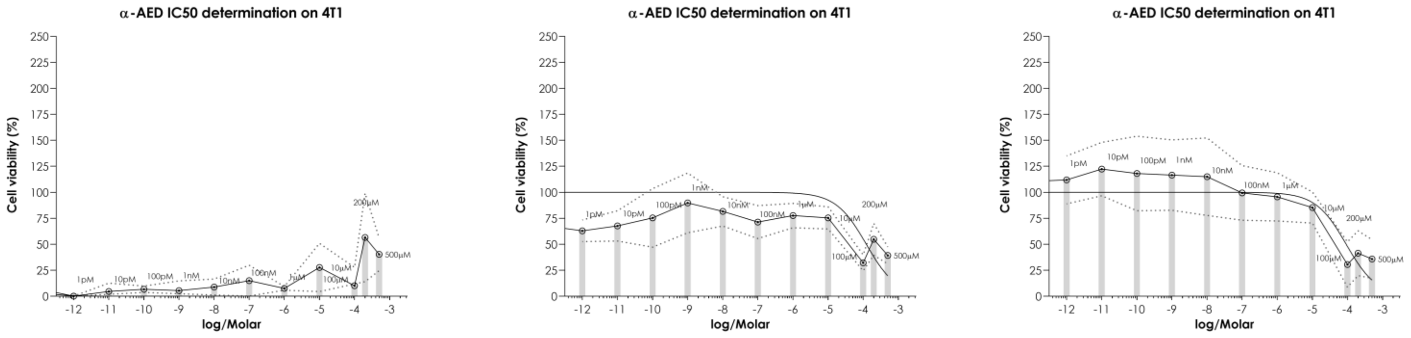

Figure S1. Effect of α-AED in 4T1 cells. The 4T1 cell line was exposed to vehicle (EtOH 0.2%) and α-AED for 24, 48, and 72 hours. Cell density was measured through SRB assay. Cell viability a as a percentage to obtain half-maximal inhibitory concentration (IC50).

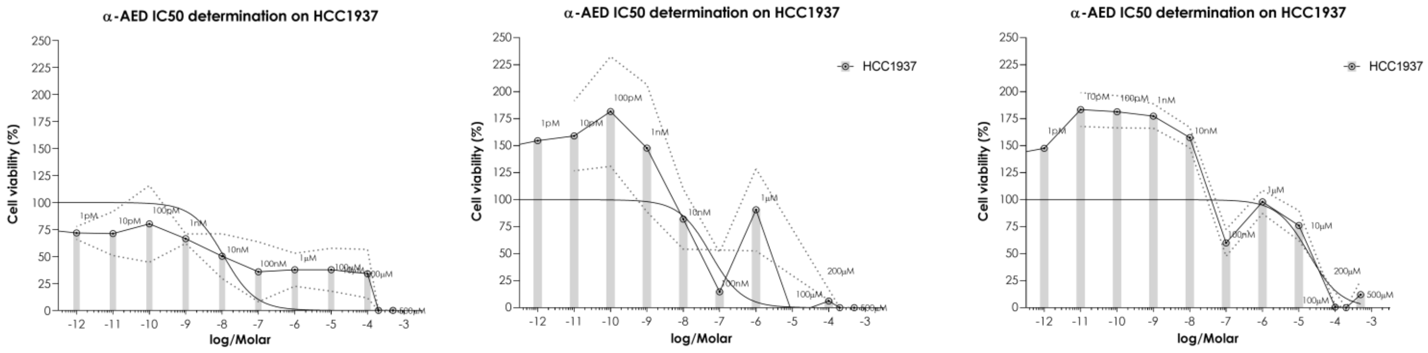

Figure S2. Effect of α-AED in HCC1937 cells. The HCC1937 cell line was exposed to vehicle (EtOH 0.2%) and α-AED for 24, 48, and 72 hours. Cell density was measured through SRB assay. Cell viability was adjusted as a percentage to obtain half-maximal inhibitory concentration (IC_{50}).
